# Standardised inventories of spiders (Arachnida, Araneae) of Macaronesia III: dry habitats of Cabo Verde Archipelago (São Vicente and Santo Antão)

**DOI:** 10.3897/BDJ.12.e115464

**Published:** 2024-03-28

**Authors:** Luís Carlos Fonseca Crespo, Pedro Cardoso, Jagoba Malumbres-Olarte, Fernando Pereira, Maria Romeiras, Alejandra Ros-Prieto, François Rigal, Paulo A. V. Borges

**Affiliations:** 1 Laboratory for Integrative Biodiversity Research (LIBRe), Finnish Museum of Natural History (LUOMUS), University of Helsinki, Pohjoinen Rautatiekatu 13, Helsinki, 00100, Finland Laboratory for Integrative Biodiversity Research (LIBRe), Finnish Museum of Natural History (LUOMUS), University of Helsinki, Pohjoinen Rautatiekatu 13 Helsinki, 00100 Finland; 2 cE3c- Centre for Ecology, Evolution and Environmental Changes/Azorean Biodiversity Group, CHANGE – Global Change and Sustainability Institute, School of Agricultural and Environmental Sciences, University of the Azores, Rua Capitão João d´Ávila, Pico da Urze, 9700-042, Angra do Heroísmo, Azores, Portugal cE3c- Centre for Ecology, Evolution and Environmental Changes/Azorean Biodiversity Group, CHANGE – Global Change and Sustainability Institute, School of Agricultural and Environmental Sciences, University of the Azores, Rua Capitão João d´Ávila, Pico da Urze, 9700-042 Angra do Heroísmo, Azores Portugal; 3 cE3c - Centre for Ecology, Evolution and Environmental Changes (cE3c) & CHANGE - Global Change and Sustainability Institute, Faculdade de Ciências, Universidade de Lisboa, Campo Grande, 1749-016, Lisbon, Portugal cE3c - Centre for Ecology, Evolution and Environmental Changes (cE3c) & CHANGE - Global Change and Sustainability Institute, Faculdade de Ciências, Universidade de Lisboa, Campo Grande, 1749-016 Lisbon Portugal; 4 LEAF—Linking Landscape, Environment, Agriculture and Food Research Center & Associated Laboratory TERRA, Instituto Superior de Agronomia (ISA), Universidade de Lisboa, Tapada da Ajuda, 1340-017, Lisbon, Portugal LEAF—Linking Landscape, Environment, Agriculture and Food Research Center & Associated Laboratory TERRA, Instituto Superior de Agronomia (ISA), Universidade de Lisboa, Tapada da Ajuda, 1340-017 Lisbon Portugal; 5 Environment and Microbiology Team, Université de Pau et des Pays de l’Amour, Pau Cedex 64013, France Environment and Microbiology Team, Université de Pau et des Pays de l’Amour Pau Cedex 64013 France; 6 IUCN SSC Atlantic Islands Invertebrate Specialist Group, 9700-042, Angra do Heroísmo, Azores, Portugal IUCN SSC Atlantic Islands Invertebrate Specialist Group, 9700-042 Angra do Heroísmo, Azores Portugal; 7 IUCN SSC Species Monitoring Specialist Group, 9700-042, Angra do Heroísmo, Azores, Portugal IUCN SSC Species Monitoring Specialist Group, 9700-042 Angra do Heroísmo, Azores Portugal

**Keywords:** Arthropoda, Atlantic Ocean, shrubland habitat, island endemics, introduced species, COBRA protocol

## Abstract

**Background:**

With this publication, we contribute to the knowledge of the arachnofauna of Cabo Verde, focusing specifically on the Islands of Santo Antão and São Vicente. Data were obtained from samples collected as part of the project "Macaronesian Islands as a testing ground to assess biodiversity drivers at multiple scales" (FCT - MACDIV, 2015-2018). This project aimed to identify the factors influencing community assembly in Macaronesian islands. For the Cabo Verde Islands, we focused on dry habitats with the additional aim to revise the aracnofauna of this poorly-known fauna. We applied the COBRA (Conservation Oriented Biodiversity Rapid Assessment) sampling protocol in ten 50 m x 50 m dry shrub plots, with five on each of the two islands, using pitfall traps, sweep-netting and active search. Additional ad-hoc samples were also collected and reported.

**New information:**

Our sampling of spiders from Cabo Verde (Santo Antão and São Vicente) yielded a total of 3,368 specimens, of which 1300 (39%) were adults. The samples include 21 families, 87 species, 18 of which are morphospecies awaiting formal identification or description at species level. Species in the families Oxyopidae (2 spp.) and Araneidae (8 spp.) were the most abundant, making up 49% of the specimens. From the 68 named species, 14 are endemic to Cabo Verde, 40 are native non-endemic and 14 are introduced. The colonisation status of *Cithaeronreimoseri* Platnick, 1991 is unknown. Endemic species accounted for 24% (n = 818) of the specimens and native non-endemic for 63% (n = 2122). A total of 29 species were new records for Cabo Verde, with 15 for Santo Antão, seven for São Vicente and seven for both Islands.

## Introduction

The Cabo Verde Archipelago is situated around 17° N and 23° W in the Atlantic Ocean, roughly 570 km west of Africa. It is composed of 10 islands spread across three groups (southern, northern and eastern). Mountains with ranges spanning from 750 m (Monte Verde, São Vicente) to 2829 m (Pico do Fogo, Fogo) are present on some of the islands, creating landscape and habitat diversity (São Vicente, Santo Antão, Fogo, Santiago, São Nicolau, Brava), while other islands are generally of lesser altitude and ecologically more homogeneous (Santa Luzia, Sal, Boa Vista, Maio). The Archipelago is amongst the oldest of Macaronesia, with the stem volcanic activity of the region dating up to 22 M.a., although the subaerial parts of present day Cabo Verde are generally younger (Holm et al. 2008).

Although historically placed in Macaronesia (Dansereau 1961), given some affinities of its highland flora with Mediterranean and Palaearctic Regions, Cabo Verde has not been considered part of this biogeographic region by several authors (Nicolás et al. 1989, Rivas-Martínez 2009). More recently, it has been argued that Macaronesia should be redefined and Cabo Verde should consitute its own biogeographic region, based on the affinities of marine taxa (Freitas et al. 2019). As with many insular ecosystems, the biota of Cabo Verde was severely affected by the arrival of humans after 1460 (Ribeiro 1955), who introduced animals and plants, irreversibly altering the Islands. It is likely that nowadays the Islands are even drier than originally, due to removal of tree cover for early settlements. This aridification process is common in Macaronesian islands, such as the Desertas, Porto Santo (in Madeira Archipelago) or the Azores. In all these cases, most endemic species are nowadays restricted to the most humid areas of the islands, while largely replaced by widespread surrogate species in drier areas. In the particular case of Macaronesian spider communities, this replacement pattern was identified several times (Borges and Wunderlich 2008, Crespo et al. 2020).

The spider fauna of Cabo Verde received little interest from pioneer arachnologists. The earliest seven endemics were described by Blackwall (Blackwall 1865), then two subsequent species by Simon (Simon 1883), followed by another seven species described by Berland (Berland 1936). It was only more than half a century later that further endemics were described in the taxonomic works of Schmidt et al. (Schmidt et al. 1994, Schmidt and Krause 1994, Schmidt and Krause 1995a, Schmidt and Krause 1995b, Schmidt and Krause 1998, Schmidt 1999, Schmidt 2001). Apart from few other additional isolated descriptions of Cabo Verde Archipelago endemic spiders or faunistic reports (Denis 1941, Jocqué 1981, [Bibr B11203789][Bibr B10462468], Baessa-de-Aguiar 1998, Assmuth and Groh 1982, Wesolowska 1989, Lotz 2011) or nomenclatural changes referring to the former studies, no recent revisions have been published. In fact, for some species, the types have never been revised, resulting in the presence of rather obscure and poorly-known endemic taxa. In some cases, the type material may be lost, as is the case of the old collections of John Blackwall. All known records were compiled in García et al. (2005), accounting for 103 species in the Archipelago, to which the citation of Devadecf.indistincta is added after a survey in the Island of Maio (Breitling et al. 2012), reaching 104 species. This poor level of knowledge might obscure unknown radiation phenomena. Nine genera of spiders currently reported for Cabo Verde present more than one species in the Archipelago, but the real number of these endemic taxa might be larger than currently known, especially considering that past and current work only sampled two of the ten islands of the Archipelago.

## General description

### Purpose

The MACDIV project aimed to advance the fundamental knowledge of the factors responsible for existing diversity patterns using the Macaronesian Islands as model systems and spiders as model organisms (Malumbres‐Olarte et al. 2021). Focusing on local scales (plots 50 m x 50 m), MACDIV intended to dissect the taxonomic, functional and evolutionary basis of spatial heterogeneity in diversity, providing opportunities to understand some of the key processes that have led to the great diversification of life in Macaronesia.

The current manuscript is the third data paper contribution to the MACDIV project. The previous manuscripts focused on the spider species from the Azores (Malumbres-Olarte et al. 2019) and Madeira (Malumbres-Olarte et al. 2020) Archipelagos.

With the current contribution, we aim to provide the raw spider distribuition and abundance data for ten studied plots in Cabo Verde Islands and to respond to the Wallacean and Prestonian shortfalls. Respectively, the former hinders the knowledge of the distribution of species, while the latter hinders the knowledge of the abundance of species and both contribute to a great difficulty of establishment conservation priorities for invertebrates (Cardoso et al. 2011).

### Additional information

The general objectives of the MACDIV project were: i) To characterise cross-scale variations of Taxonomic Diversity (TD), Functional Diversity (FD) and Phylogenetic Diversity (PD) from plot to archipelago scales; ii) to identity the spatial, historical and environmental factors that may influence TD, FD and PD patterns at local scales; and iii) to determine resource-use differentiation between species to quantify the importance of ecological interactions within communities in driving local patterns.

## Project description

### Title

Standardised inventories of spiders (Arachnida, Araneae) of dry habitats of Cabo Verde Archipelago (São Vicente and Santo Antão Islands)

### Personnel

The project was conceived and led by Paulo A.V. Borges, François Rigal and Pedro Cardoso.

Fieldwork (site selection and experimental setting): Maria Romeiras, Paulo A.V. Borges and Pedro Cardoso.

Fieldwork (authorisation): Direcção Nacional do Ambiente de Cabo Verde; INIDA – Instituto Nacional de Investigação e Desenvolvimento Agrário de Cabo Verde.

**Figure 1.**
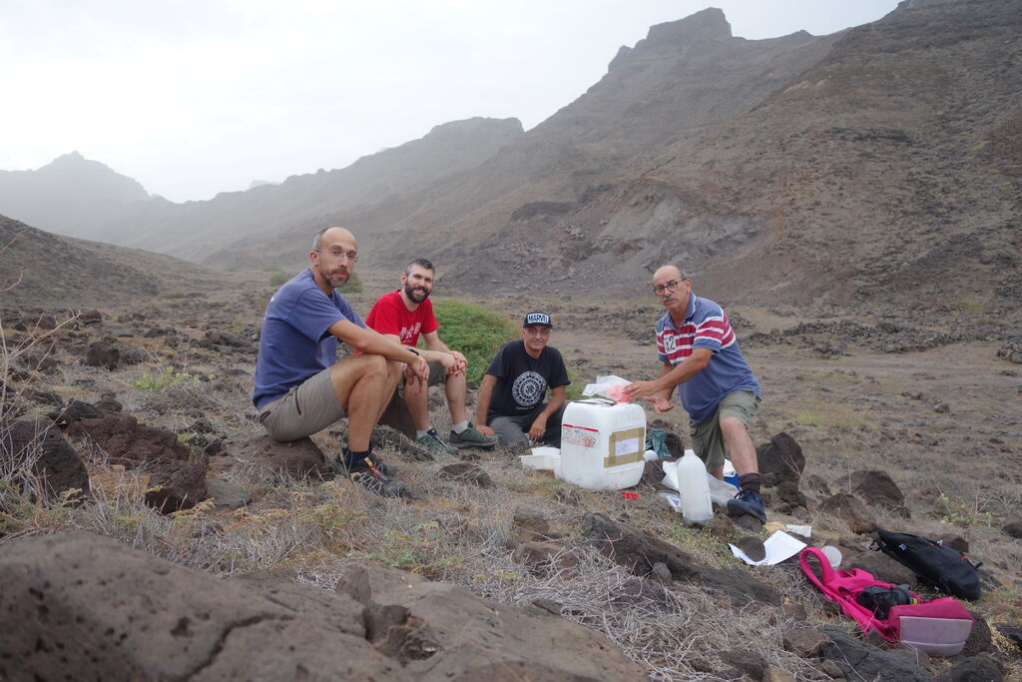
The fieldwork team: Pedro Cardoso, Jagoba Malumbres-Olarte, Paulo A. V. Borges and Fernando Pereira (Credit: Pedro Cardoso).

Fieldwork (spider sampling): Paulo A. V. Borges, Pedro Cardoso, Jagoba Malumbres-Olarte and Fernando Pereira (Fig. 1).

Parataxonomists: Alejandra Ros-Prieto and Paulo A. V. Borges.

Taxonomist: Luís Carlos Crespo.

Voucher specimen management was undertaken by Alejandra Ros Prieto and Paulo A. V. Borges.

Database management was undertaken by Alejandra Ros Prieto and Paulo A. V. Borges.

Darwin Core databases: Paulo A. V. Borges and Luís Carlos Crespo.

### Study area description

The Macaronesian Islands comprise four main archipelagos (Azores; Madeira and Selvagens; Canary Islands; Cabo Verde), with 40 islands larger than 1 km^2^ covering a latitudinal range from 14.8°N to 39.7°N (Florencio et al. 2021).

Cabo Verde is an archipelago located in the central Atlantic Ocean composed of a group of volcanic islands and islets, ten main islands and several smaller islets and rocks. The islands are divided into three groups: northern (Santo Antão, São Vicente, Santa Luzia and São Nicolau), southern (Brava, Fogo and Santiago) and eastern (Maio, Boa Vista and Sal). The Archipelago is of volcanic origin and many of them still exhibit volcanic landscapes, including craters, lava flows and rugged terrain. The geography of Cabo Verde Islands is diverse. Some islands have lush, green landscapes with mountain ranges, while others are more arid and characterised by semi-desert conditions. Santo Antão, for example, is known for its dramatic mountainous terrain, while Boa Vista and Maio are flatter and have sand dunes. The Islands experience a tropical climate with distinct wet and dry seasons. The northern Islands receive more rainfall and tend to be greener, while the southern Islands are generally drier.

The geomorphological characteristics of our focal Islands of Santo Antão and São Vicente are marked by great ecological and edaphic diversity, similar to the other mountainous Islands of the Cabo Verde Archipelago (Santiago, Fogo, Brava and São Nicolau). The Island of Santo Antão has several volcanic cones, the highest point being Tope de Coroa (1980m). On the slopes facing the north-eastern trade winds, the erosion that has taken place over the course of the Island's evolution has been intense, shaping the landscape with several streams (e.g. Ribeira da Torre, Ribeira da Garça, Ribeira do Paúl, Ribeira da Janela) characterised by a sub-humid climate. Particularly, in the high-altitude valleys, a rich endemic flora can be found (e.g. *Campylanthusglaber*, *Diplotaxisgorgadensis*, Echiumstenosiphonsubsp.lindbergii, *Phagnalonmelanoleucum*, *Sonchusdaltonii*, *Tolpisfarinulosa*, *Umbilicus schmidtii*). Other mountainous regions, such as Pico da Cruz (1585 m) and Planalto Leste (located in the Cova/Paul/Ribeira da Torre Natural Park), also show a great floristic diversity, including endemic plants together with exotic trees (e.g. *Pinuscanariensis*, *Eucalyptus* and *Cupressus*) which constitute one of the most important forest resources of this Archipelago.

In the north-facing coastal cliffs of Santo Antão, characterised by temporary river valleys and escarpments, other endemic flora can still be found (e.g. *Asparagussquarrosus*, *Cynanchumdaltonii*, *Limoniumbraunii*, *Paronychiaillecebroides*, *Polycarpaeagayi*). On the southern, western and south-western slopes, the climate is more arid and the landscape is characterised by xerophytic species with sparse cover and a lack of endemic species (e.g. *Abutilonpannosum*, *Calotropisprocera*, *Cenchrusciliaris* and *Cleomeviscosa*).

São Vicente Island is very arid, but above 400 m, the influence of the north-east trade winds in the main mountainous areas (i.e. Monte Verde - 750 m and Madeiral - 680 m) creates suitable ecological conditions for the survival of some endemic plant species (e.g. *Aeoniumgorgoneum*, *Diplotaxisvogelli*, Echiumstenosiphonsubsp.stenosiphon, *Limoniumjovibarba*, Lobulariacanariensissubsp.spathulata). The lowland areas are covered by a very sparse herbaceous vegetation and the native vegetation is often heavily degraded and affected by overgrazing. Further, the high rate of invasion of the exotic tree - *Prosopisjuliflora* (synonymous with *Neltumajuliflora*), has led to a reduction of other native trees, such as *Tamarixsenegalensis*. On the north-eastern and eastern coastal areas of São Vicente Island, there are important plant communities, mainly associated with dunes and beach areas (e.g. *Cistanchephelypaea*, *Patellifoliaprocumbens*, *Polycarpaeacaboverdeana*, *Suaedacaboverdeana*, *Zygophyllumfontanesii*) and salt marshes (e.g. Frankeniaericifoliasubsp.caboverdeana, *Patellifoliaprocumbens*, *Sesuviumportulacastrum* and Zygophyllumgaetulumsubsp.waterlotii).

### Design description

**Figure 2.**
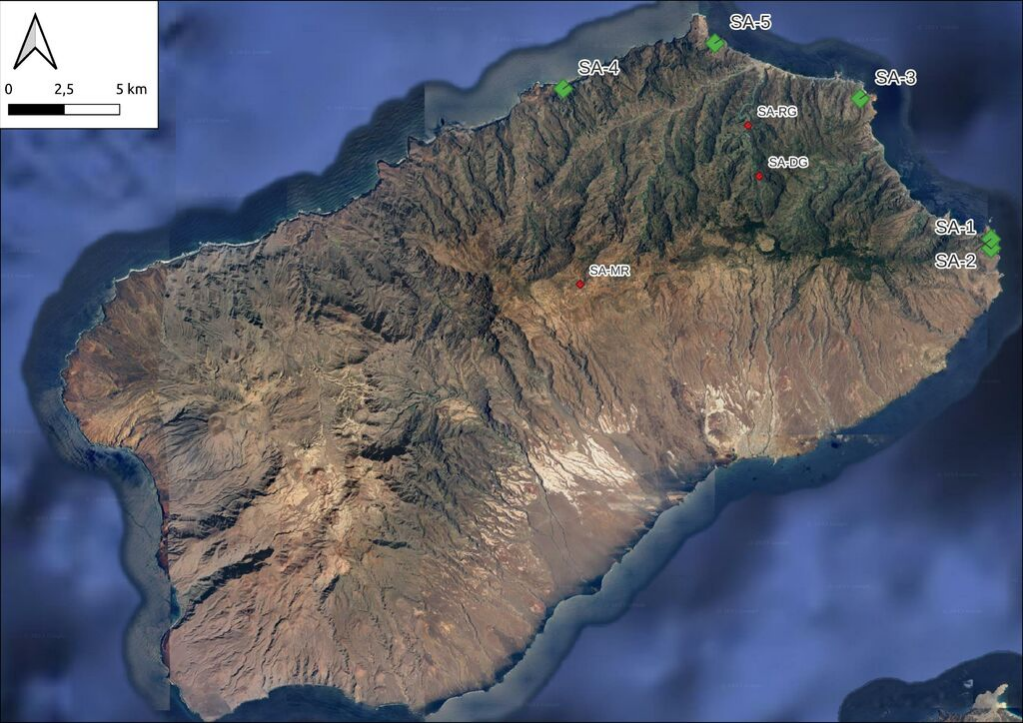
Map of Santo Antão, with the five main plots represented by larger green diamonds and the ad-hoc sites represented as smaller red diamonds. Codes as in Table 1 (Credit: Sébastien Lhoumeau).

**Figure 3.**
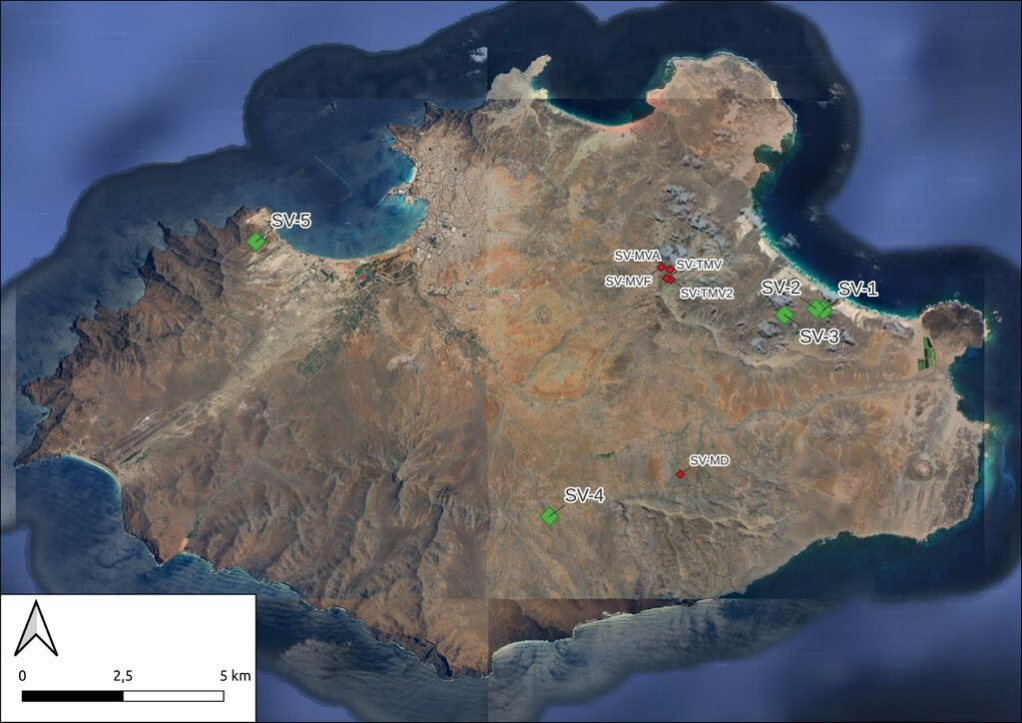
Map of São Vicente, with the five main plots represented by green diamonds and the ad-hoc sites represented as red diamonds. Codes as in Table 1 (Credit: Sébastien Lhoumeau).

Five sampling plots of 50 m x 50 m were installed in each of the two Islands (Santo Antão (Fig. 2) and São Vicente (Fig. 3)) at increasing distances from a reference plot (P1 at point 0, P2 at 0.1 km from P1, P3 at 1 km from P1, P4 at 5 km from P1 and P5 at 10 km from P1) in order to test beta-diversity models, as described in Malumbres‐Olarte et al. (2021) (Table 1).

All plots were located in areas where the habitat is arid and dominated by xerophytic community assemblages. In Santo Antão, the following plant species were present in the plots: *Abutilonpannosum*, *Aristidaadscensionis*, *Aristidafuniculata*, *Asparagussquarrosus*, Asteriscusdaltoniisubsp.vogelli, *Blainvilleagayana*, *Calotropisprocera*, Campylanthusglabersubsp.spathulatus, *Cenchrusciliaris*, *Cleomeviscosa*, *Commicarpushelenae*, *Crotalariasenegalensis*, *Cynanchumdaltonii*, Frankeniaericifoliasubsp.ericifolia, *Lavandulacoronopifolia*, *Limoniumbraunii*, *Paronychiaillecebroides*, *Polycarpaeagayi*, *Salviaaegyptiaca*, *Sesuviumportulacastrum*, *Trichodesmaafricanum* and *Zygophyllumcreticum*.

In São Vicente, the following plant species were present in the plots: *Abutilonpannosum*, *Aervajavanica*, *Andrachnetelephioides*, *Aristidaadscensionis*, *Asparagussquarrosus*, *Cleomeviscosa*, *Commicarpushelenae*, *Cynodondactylon*, *Fagoniacretica*, *Zygophyllumcreticum*, Frankeniaericifoliasubsp.ericifolia, *Gymnocarpossclerocephalus*, *Indigofera cordifólia*, *Limoniumbrunneri*, *Lotusalianus*, *Patellifoliaprocumbens*, *Polycarpaeagayi*, *Sesuviumportulacastrum*, *Suaedacaboverdeana*, *Tragusracemosus*, *Urochloacabo-verdiana*, *Zygophyllumfontanesii* and *Zygophyllumsimplex*.

**Figure 5.**
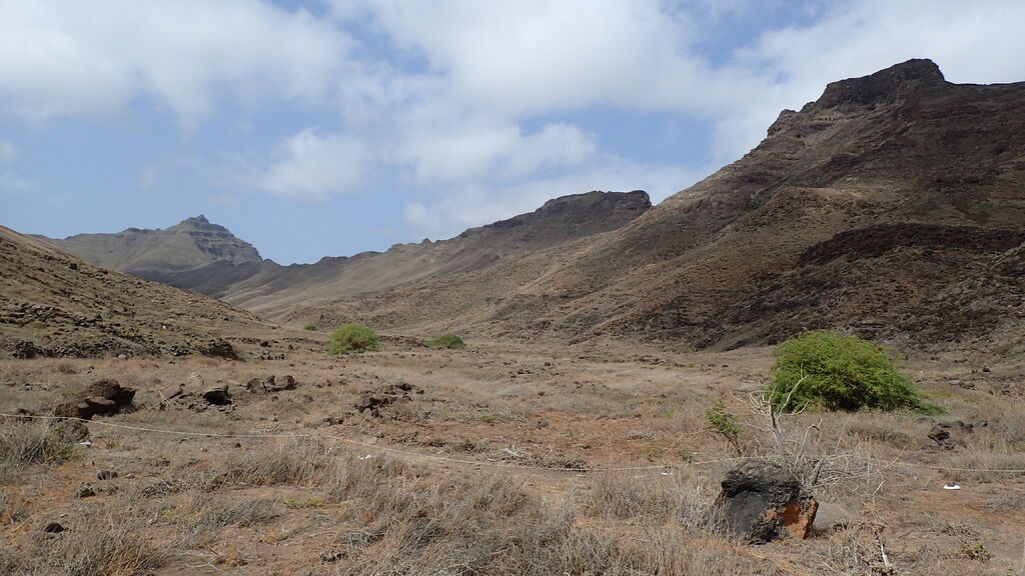
São Vicente Plot 1 - Feijoal Preto 1 (Credit: Pedro Cardoso).

The sites in Santo Antão were located in a complex orographic terrain (Fig. 4a), often close to steep slopes (Fig. 4b), whereas the plots in São Vicente were mostly located in flat terrain (Fig. 5).

### Funding

Fieldwork and initial research (2015-2018): FCT MACDIV (Macaronesian Islands as a testing ground to assess biodiversity drivers at multiple scales) was supported by Fundação para a Ciência e a Tecnologia – ref. FCT-PTDC/BIABIC/0054/2014.

Additional taxonomic work (2023): FCT-UIDB/00329/2020-2024 (Thematic Line 1 – integrated ecological assessment of environmental change on biodiversity).

## Sampling methods

### Study extent

To assess spider diversity, we employed the COBRA (Conservation Oriented Biodiversity Rapid Assessment) sampling protocol for habitats with no arboreal vegetation (Cardoso 2009). This protocol was later adapted to be the standard protocol to survey and monitor island communities (Borges et al. 2018). We employed the following sampling regime:

**Vegetation sweeping (SW)**: We used a sweep-net with a 46 cm diameter opening for sweeping shrubs and tall herbaceous plants. A sweep-net sample consisted of one hour of sweeping, including the search for displaced spiders (Fig. 6a). The sampling protocol included four daytime (SWD) and four nighttime (SWN) samples, all performed at similar hours. We stored the specimens collected by this method in vials with 96% alcohol.

**Night-time active aerial searching (AAS)**: Involved the systematic collection of spiders located above knee-level through manual, forceps, pooter or brush-assisted methods. We stored the specimens collected by this method as for the sweeping sampling. We did four samples of one hour per plot at similar hours.

**Pitfall (PIT) Method**: A total of 48 pitfall traps, which adhere to the European standard of 8 cm width at the top and a depth of approximately 12 cm (using 33 cl plastic cups), were strategically positioned just outside the boundary of each sampling plot. Traps were distributed evenly (around 4 m from each other), with 12 placed along each side of the square plot. They were 33% filled with 100% propylene glycol and left undisturbed in the field for a period of 14 days. To safeguard against predation, rainwater inundation and the capture of unwanted vertebrates (such as reptiles), plastic plates were positioned on stilts or rocks approximately 2 cm above the ground surface (Fig. 6b). In terms of data analyses, a group of four consecutive traps was considered as a single sample, totalling 12 samples per plot. However, in the current database, the individual 48 pitfall data are available (Note: pitfall samples missing in the event table are samples in which no spiders were collected).

### Quality control

In the laboratory, specimen sorting and spider identification followed standard procedures, using somatic and genitalic features for species identification, with literature available from the World Spider Catalogue (World Spider Catalogue 2023). Normally, juveniles are not identified to species level, but given the relative low number of species in insular faunas, more often than not we could match a juvenile to an adult, based on somatic resemblance. For this reason and to be consistent with previous publications (Malumbres-Olarte et al. 2019, Malumbres-Olarte et al. 2020), we considered juvenile specimens in abundance counts. A reference collection was made for all collected specimens (whether or not identified at the species level) by assigning them a morphospecies code number and deposited at the Dalberto Teixeira Pombo Insect Collection (DTP), University of Azores (Terceira Island, Portugal).

## Geographic coverage

### Description

Cabo Verde Islands of Santo Antão and São Vicente.

### Coordinates

16.76772 and 17.210328 Latitude; -25.38116 and -24.83459 Longitude.

## Taxonomic coverage

### Description

All spiders (Arachnida, Araneae). The samples include 21 families.

### Taxa included

**Table:** 

Rank	Scientific Name	Common Name
order	Araneae	Spiders

## Temporal coverage

**Data range:** 2017-10-30 – 2017-11-23.

### Notes

The sampling occurred during a field expedition to the Islands of Santo Antão and São Vicente in October-November 2017. Sites were selected with the collaboration of local experts and the main fieldwork occurred between 30 October and 12 November. In the period between 14 and 23 November, pitfall traps were recovered.

## Collection data

### Collection name

Dalberto Teixeira Pombo insect collection at the University of Azores.

### Collection identifier

DTP

### Specimen preservation method

All specimens were preserved in 96% ethanol.

### Curatorial unit

Dalberto Teixeira Pombo insect collection at the University of Azores (Curator: Paulo A. V. Borges).

## Usage licence

### Usage licence

Creative Commons Public Domain Waiver (CC-Zero)

## Data resources

### Data package title

Spiders from Macaronesia: Cabo Verde

### Resource link

https://doi.org/10.15468/p94j28

### Alternative identifiers

https://www.gbif.org/dataset/b8eeccd9-52dd-4e43-949e-818402577fa8; http://ipt.gbif.pt/ipt/resource?r=spiders_cabo_verde_2023

### Number of data sets

2

### Data set 1.

#### Data set name

Event Table

#### Data format

Darwin Core Archive format

#### Character set

UTF-8

#### Download URL

http://ipt.gbif.pt/ipt/resource?r=spiders_cabo_verde_2023

#### Data format version

1.10

#### Description

The dataset was published in the Global Biodiversity Information Facility platform, GBIF (Borges et al. 2023). The following data table includes all the records for which a taxonomic identification of the species was possible. The dataset submitted to GBIF is structured as a sample event dataset that has been published as a Darwin Core Archive (DwCA), which is a standardised format for sharing biodiversity data as a set of one or more data tables. The core data file contains 382 records (eventID). This GBIF IPT (Integrated Publishing Toolkit, Version 2.5.6) archives the data and, thus, serves as the data repository.

**Data set 1.** 

Column label	Column description
id	Identifier of the event.
eventID	Identifier of the events, unique for the dataset. The first three letters refer to the island (STA- Santo Antão; VIC - São Vicente) and the following codification includes plot number, sampling method and sample number.
samplingProtocol	The names of, references to, or descriptions of the methods or protocols used during a dwc:Event. Six possible sampling methods are listed: Direct search Day; Direct search Night; Active Aerial Search Night; Sweeping Day; Sweeping Night; Pitfall.
sampleSizeValue	A numeric value for a measurement of the size (time duration, length, area or volume) of a sample in a sampling dwc:Event.
sampleSizeunit	The unit of measurement of the size (time duration, length, area or volume) of a sample in a sampling dwc:Event.
samplingEffort	The amount of effort expended during a dwc:Event.
eventDate	The date-time or interval (in case of Pitfall trapping) during which a dwc:Event occurred.
year	The four-digit year in which the dwc:Event occurred, according to the Common Era Calendar.
month	The integer month in which the dwc:Event occurred.
day	The integer day of the month on which the dwc:Event occurred.
habitat	A category or description of the habitat in which the dwc:Event occurred. In our case, the single surveyed habitat was dry shrubland.
fieldNumber	An identifier given to the event in the field. In the case of the plots, codification includes the number of the plot, a code for the sampling protocol and sample number.
islandGroup	The name of the island group in which the dcterms:Location occurs, in this case, Cabo Verde.
island	The name of the island on or near which the dcterms:Location occurs.
country	The name of the country or major administrative unit in which the dcterms:Location occurs, in this case, Cabo Verde.
countryCode	The standard code for the country in which the dcterms:Location occurs (CV).
stateProvince	The name of the next smaller administrative region than country (state, province, canton, department, region etc.) in which the dcterms:Location occurs.
minimumElevationInMeters	The lower limit of the range of elevation (altitude, usually above sea level), in meters.
locationRemarks	Comments or notes about the dcterms:Location. In this case we put the names of localities in ad-hoc samples or Plot number.
locality	The original textual description of the place.
decimalLatitude	The geographic latitude (in decimal degrees, using the spatial reference system given in dwc:geodeticDatum) of the geographic centre of a dcterms:Location. Positive values are north of the Equator, negative values are south of it. Legal values lie between -90 and 90, inclusive.
decimalLongitude	The geographic longitude (in decimal degrees, using the spatial reference system given in dwc:geodeticDatum) of the geographic centre of a dcterms:Location. Positive values are east of the Greenwich Meridian, negative values are west of it. Legal values lie between -180 and 180, inclusive.
geodeticDatum	The ellipsoid, geodetic datum or spatial reference system (SRS) upon which the geographic coordinates given in dwc:decimalLatitude and dwc:decimalLongitude are based.
coordinateUncertaintyInMeters	The horizontal distance (in meters) from the given dwc:decimalLatitude and dwc:decimalLongitude describing the smallest circle containing the whole of the dcterms:Location. Leave the value empty if the uncertainty is unknown, cannot be estimated or is not applicable (because there are no coordinates). Zero is not a valid value for this term.
coordinatePrecision	A decimal representation of the precision of the coordinates given in the dwc:decimalLatitude and dwc:decimalLongitude.
georeferenceSources	A list (concatenated and separated) of maps, gazetteers or other resources used to georeference the dcterms:Location, described specifically enough to allow anyone in the future to use the same resources.

### Data set 2.

#### Data set name

Occurrence Table

#### Data format

Darwin Core Archive format

#### Character set

UTF-8

#### Data format version

1.10

#### Description

The dataset was published in the Global Biodiversity Information Facility platform, GBIF (Borges et al. 2023). The following data table includes all the records for which a taxonomic identification of the species was possible. The dataset submitted to GBIF is structured as an occurrence table that has been published as a Darwin Core Archive (DwCA), which is a standardised format for sharing biodiversity data as a set of one or more data tables. The core data file contains 982 records (occurrenceID). This GBIF IPT (Integrated Publishing Toolkit, Version 2.5.6) archives the data and, thus, serves as the data repository.

**Data set 2.** 

Column label	Column description
id	Identifier of the event.
type	The nature or genre of the resource, in this case "PhysicalObject".
licence	Information about rights held in and over the resource.
institutionID	An identifier for the institution having custody of the object(s) or information referred to in the record.
collectionID	An identifier for the collection or dataset from which the record was derived.
institutionCode	The name (or acronym) in use by the institution having custody of the object(s) or information referred to in the record (UAc - Universidade dos Açores).
collectionCode	The name, acronym, coden or initialism identifying the collection or dataset from which the record was derived (DTP - Dalberto Teixeira Pombo).
datasetName	The name identifying the dataset from which the record was derived (MACDIV).
basisOfRecord	The specific nature of the data record (PreservedSpecimen).
occurrenceID	An identifier for the dwc:Occurrence (as opposed to a particular digital record of the dwc:Occurrence). In the absence of a persistent global unique identifier, construct one from a combination of identifiers in the record that will most closely make the dwc:occurrenceID globally unique.
recordedBy	A list (concatenated and separated) of names of people, groups or organisations responsible for recording the original dwc:Occurrence. The primary collector or observer, especially one who applies a personal identifier (dwc:recordNumber), should be listed first.
organismQuantity	A number or enumeration value for the quantity of dwc:Organisms.
organismQuantityType	The type of quantification system used for the quantity of dwc:Organisms.
sex	The sex of the biological individual(s) represented in the dwc:Occurrence.
lifeStage	The age class or life stage of the dwc:Organism(s) at the time the dwc:Occurrence was recorded.
establishmentMeans	The process of establishment of the species in the location, using a controlled vocabulary: 'endemic', 'native', 'introduced', 'indeterminate'.
eventID	Identifier of the events, unique for the dataset. The first three letters refer to the Island (STA- Santo Antão; VIC - São Vicente) and the following codification includes plot number, sampling method and sample number.
identifiedBy	A list (concatenated and separated) of names of people, groups or organisations who assigned the dwc:Taxon to the subject.
dateIdentified	The date on which the subject was determined as representing the dwc:Taxon.
identificationRemarks	Comments or notes about the dwc:Identification.
scientificName	The full scientific name, with authorship and date information, if known. When forming part of a dwc:Identification, this should be the name in the lowest level taxonomic rank that can be determined. This term should not contain identification qualifications, which should instead be supplied in the dwc:identificationQualifier term.
kingdom	The full scientific name of the kingdom in which the dwc:Taxon is classified.
phylum	The full scientific name of the phylum or division in which the dwc:Taxon is classified.
class	The full scientific name of the class in which the dwc:Taxon is classified.
order	The full scientific name of the order in which the dwc:Taxon is classified.
family	The full scientific name of the family in which the dwc:Taxon is classified.
genus	The full scientific name of the genus in which the dwc:Taxon is classified.
specificEpithet	The name of the first or species epithet of the dwc:scientificName.
taxonRank	The taxonomic rank of the most specific name in the dwc:scientificName.
scientificNameAuthorship	The authorship information for the dwc:scientificName formatted according to the conventions of the applicable dwc:nomenclaturalCode.

## Additional information

The collected samples generated a total of 3,361 specimens, amongst which 1300 (39%) were adults. The samples include 21 families, 65 genera and 87 species (Table 2). Species from the Oxyopidae and Araneidae families were the most abundant, comprising two and eight species, respectively, making up 49% of the specimens of the identified taxa. From the 69 identified species, 14 are endemic to Cabo Verde, 40 native non-endemic and 14 exotic introduced species. For *Cithaeronreimoseri*, the colonisation status was indeterminate. Endemic species accounted to 24% (n = 818) of the specimens and native to 63% (n = 2122). Introduced species accounted for 208 specimens. Most of the exotic introduced species were not abundant with the clear exception of *Modisimusculicinus* (Simon, 1893) (see Table 2).

**Figure 7.**
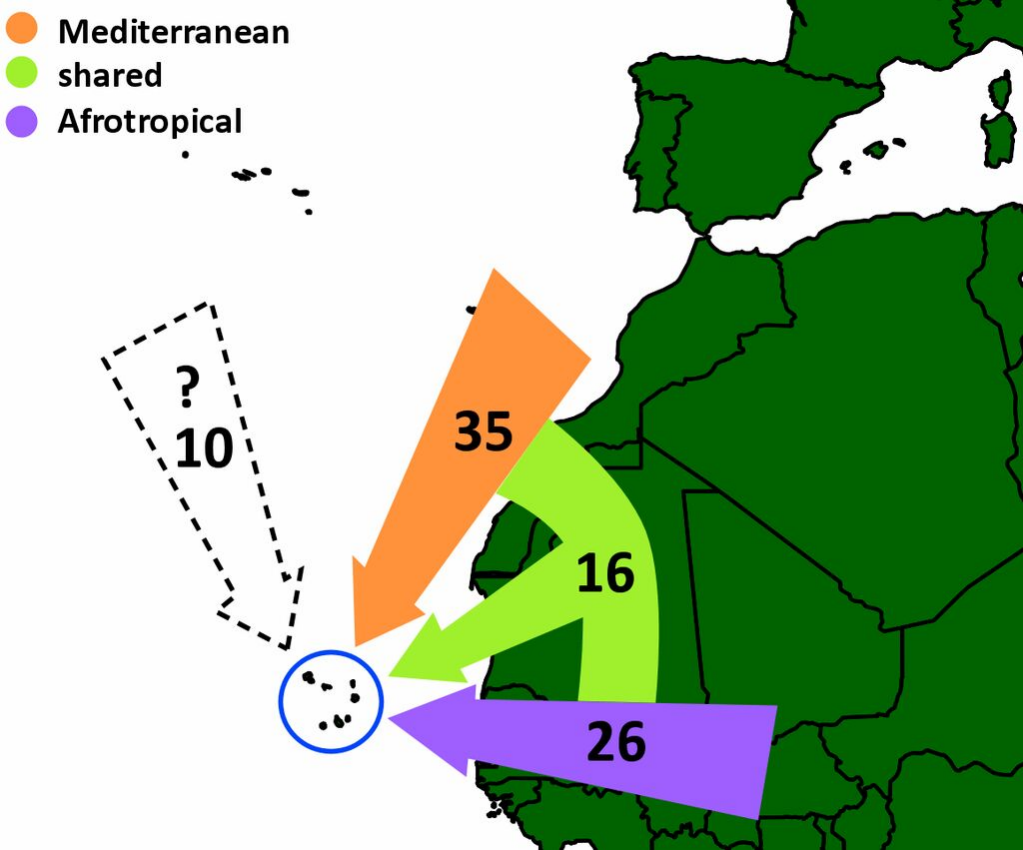
Biogeographic affinities of the collected species. We have categorised species as Mediterranean, Afrotropical, present in both regions or with an unknown biogeographical affinity. For unidentified species, we have pondered if the genus or genera-group are predominantly present in one of the regions. Known introductions are taken according to what is known from the World Spider Catalogue and are treated as unknown.

We present the biogeographic affinities of the collected species (Fig. 7), which show a higher prevalence of Mediterranean taxa compared with Afrotropical taxa. This suggests that the arid to semi-arid habitats encountered in most of the Cabo Verde Archipelago might favour common taxa in the Mediterranean against common taxa in the Afrotropics.

### Taxonomic remarks

A total of 29 species were new records for Cabo Verde, being 15 new records for only Santo Antão, seven new records for only São Vicente and seven species were new records for both Islands. A total of 18 species were potentially new species to science or still undetermined members of previously-described taxa.

Given the abundance of new findings to the Cabo Verde Archipelago araneofauna, below we highlight the 29 cases of new records to the Archipelago, alongside the 18 unidentified species, for each commenting on their known distribution ranges, according to what is known from the WSC (World Spider Catalogue 2023).

**Family Araneidae Clerck, 1757**

***Cyphalonotus* CV89**: This morphospecies comprises a likely undescribed species. It is not an Archipelago endemic, being also present on mainland Africa (Ghana), as identified by the first author from the collections at the Finnish Museum of Natural History. It was previously only known to us from undescribed males, with additional records here for the Archipelago from a male and 6 juveniles.

***Neosconaquincasea* Roberts, 1983**: New record for the Archipelago. Widespread in the Afrotropics. Only two adult male specimens were newly collected, one in each Island.

**Family Cheiracanthiidae Wagner, 1887**

***Cheiracanthiumafricanum* Lessert, 1921**: New record for the Archipelago and found from both adult males and females only in Santo Antão. Widespread in the Afrotropics.

***Cheiracanthium* CV6**: We believe the genus *Cheiracanthium* needs to be revised carefully for the Cabo Verde Islands. The endemic species *C.halophilum* Schmidt & Piepho, 1994 is problematic as it might represent more than one species. It was described, based on females from Boa Vista Island (including the holotype) and males from both São Vicente and Maio Islands, plus subsequently an additional male from Santo Antão (Lotz 2007). The present morphospecies, *Cheiracanthium* CV6, was found exclusively in Santo Antão and, while the male matches with paratype males of *C.halophilum*, the female does not. A second endemic species of this genus, *C.verdense* Lotz, 2011 is known exclusively from males from São Vicente Island. We have found additional adult males and attribute several adult females collected alongside the males to it (the female remains undescribed).

**Family Cithaeronidae Simon, 1893**

***Cithaeronreimoseri* Platnick, 1991**: This obscure species has a disjunct distribution with an original record from Eritrea, but later also from Brazil - where it is supposedly introduced, since the genus occurs naturally in Africa up to Asia. Its presence in Cabo Verde suggests this species might occur throughout the Afrotropics. New record for the Archipelago and found in São Vicente only.

**Family Dictynidae O. Pickard-Cambridge, 1871**

***Dictyna* CV4**: This morphospecies comprises an unidentified species of uncertain genus, most likely *Dictyna*. Since there are no previous records of the genus *Dictyna* for Cabo Verde, this will be, at least, a new record for the Archipelago. The absence of revisionary works of Afrotropical Dictynidae limits the further identification of this morphospecies

**Family Gnaphosidae Banks, 1892**

***Camillinacordifera* (Tullgren, 1910)**: New record for the Archipelago and found only in Santo Antão. Widespread in the Afrotropics.

***Marinarozeloteslyonneti* (Audouin, 1826)**: New record for the Archipelago and found in both Islands. Widespread across the Macaronesia, Mediterranean up to Central Asia and introduced in the New World.

***Micariaignea* O. Pickard-Cambridge, 1872**: New record for the Archipelago and found in both Islands, being particularly abundant. Widespread across the Mediterranean, this is the southernmost record so far.

***Poecilochroasenilis* (O. Pickard-Cambridge, 1872)**: New record for the Archipelago and found only in São Vicente. Widespread across the Mediterranean, this is the southernmost record so far.

***Smionialineatipes* (Purcell, 1908)**: New record for the Archipelago and found only in Santo Antão. Known from three southern African countries. It probably occurs across the entire Afrotropics.

***Synaphosussyntheticus* (Chamberlin, 1924)**: New record for the Archipelago and found only in Santo Antão. Known from the southern Mediterranean and supposedly introduced in the New World. This is the southernmost record for this species.

**Family Linyphiidae Blackwall, 1859**

***Agynetafuscipalpa* (C. L. Koch, 1836)**: New record for the Archipelago and found only in Santo Antão. Widespread from Macaronesia up to Central Asia.

***Lepthyphantes* CV69**: We are unsure about this genus identification. It is probably not a *Lepthyphantes* *sensu stricto*, but a related genus of this rather diverse group of spiders that all share many morphological traits.

***Metaleptyphantesperexiguus* (Simon & Fage, 1922)**: New record for the Archipelago and found only in Santo Antão. Widespread in the Afrotropics.

**Family Oecobiidae Blackwall, 1862**

***Oecobiusmarathaus* Tikader, 1962**: New record for the Archipelago and found only in São Vicente. Widespread in the Afrotropics and supposedly introduced in the New World and Asia.

***Oecobiuspasteuri* Berland & Millot, 1940**: New record for the Archipelago and found only in Santo Antão. It presents a west African distribution.

***Oecobiussimilis* Kulczynski, 1909**: New record for the Archipelago and found in both Islands. It presents a Macaronesian distribution, also reaching St. Helena.

**Family Oonopidae Simon, 1890**

**Oonopidae CV103**: This morphospecies comprises adult male and female specimens and belongs to an undescribed genus also found in other Afrotropical locations, according to unpublished information gathered by the first author.

**Oonopidae CV172**: This morphospecies comprises a singleton adult female, which we could not place in any known genera. There are many undescribed oonopid genera in the Afrotropics.

***Opopaeaconcolor* (Blackwall, 1859)**: New record for the Archipelago and found only in Santo Antão. It is widespread in the Afrotropics.

***Opopaeadeserticola* Simon, 1892**: New record for the Archipelago and found on both Islands. This species has not been recorded yet in the Afrotropics, is supposedly native for Asia, but was also found as a supposed introduction in the New World and some Pacific islands.

***Opopaeaspeciosa* (Lawrence, 1952)**: New record for the Archipelago and found only in São Vicente. The present record along with its disjunct distribution, in South Africa and Yemen, suggest a wider presence across the Afrotropics.

***Opopaea* CV193**: This morphospecies comprises an unidentified species of which only a singleton male was found, with genital characters which seem to differentiate it from the remaining species.

***Orchestina* CV9**: *O.pavesii* (Simon, 1873) was previously cited for Cabo Verde. We found specimens very similar to it, but with small differences in the male genitalia. Other closely-related species to *O.pavesii* are elsewhere differentiated by such minor differences and we tentatively attribute these specimens to a new species.

***Xestaspisparmata* Thorell, 1890**: New record for the Archipelago and found only in Santo Antão. This species is native to Asia and supposedly introduced in both the New World and the Afrotropics.

**Family Philodromidae Thorell, 1869**

***Tibellusnigeriensis* Millot, 1942**: New record for the Archipelago and found only in Santo Antão. It is only the second known location for this species, which was only known from Mali.

**Family Pholcidae C. L. Koch, 1850**

***Modisimusculicinus* (Simon, 1893)**: New record for the Archipelago and found only in Santo Antão. Native from South America, it is an introduction in many other locations, from Europe, Africa, Asia and Australia to the Pacific Islands.

**Family Prodidomidae Simon, 1884**

***Eleleisluderitz* Rodrigues & Rheims 2020**: New record for the Archipelago and found only in São Vicente. Only known from Namibia.

***Prodidomus* CV34**: This morphospecies comprises a single, non-identified male from the Island of Santo Antão. African species of *Prodidomus* do not have recent revisions available.

***Prodidomus* CV134**: This morphospecies comprises several females from the Island of São Vicente. We could tentatively assign them to the previous male, but the possibility of both being sibling endemics makes it more reasonable to leave them as separate species for now.

**Family Salticidae Blackwall, 1841**

***Langona* CV158**: This morphospecies comprises a single unidentified female from São Vicente. *Langona* is widespread across the Afrotropics and the Sahel with 45 species (World Spider Catalogue 2023), but the non-revised status of many species hinders the identification of single females.

***Langona* CV194**: Same as above.

***Phlegra* CV195**: This morphospecies comprises a single unidentified female from São Vicente. Although initially identified as *P.bifurcata*, it is probably another species.

***Wesolowskana* CV190**: This morphospecies comprises a single female from the Island of Santo Antão, showing great resemblance to the two known species of this endemic genus of Cabo Verde. We believe this can be an additional third species of this genus.

**Family Scytodidae Blackwall, 1864**

***Scytodesunivittata* Simon, 1882**: New record for the Archipelago and found only in Santo Antão. Native to eastern Mediterranean up to India, it is an introduction in the New World and also Canary Islands.

**Family Tetrablemmidae O. Pickard-Cambridge, 1873**

***Tetrablemma* CV31**: This morphospecies comprises an unidentified species. This is the first record of a tetrablemmid for Cabo Verde, collected in both the Islands of Santo Antão and São Vicente. This family of minute spiders is not revised in the Afrotropical Region.

**Family Theridiidae Sundevall, 1833**

***Enoplognathadiversa* (Blackwall, 1859)**: New record for the Archipelago and found only in Santo Antão. Native to the Mediterranean.

***Euryopisepisinoides* (Walckenaer, 1847)**: New record for the Archipelago and found only in São Vicente. Native to the Mediterranean, it is also an introduction in South Africa, Reunion, India and China.

***Eurypoenatuberosa* (Wunderlich, 1987)**: New record for the Archipelago found on both Islands. Previously described from the Canary Islands.

***Lasaeola* CV64**: An unidentified species of this genus of tiny spiders, which are present from the New World to Asia.

***Steatodaerigoniformis* (O. Pickard-Cambridge, 1872)**: New record for the Archipelago and found only in São Vicente. Native to the eastern Mediterranean up to Japan, it is an introduction in the New World and South Africa.

***Theridion* CV39**: This morphospecies comprises an unidentified species of this very large genus. Males and females were collected from the Island of Santo Antão.

***Theridion* CV178**: This morphospecies comprises an unidentified species of this very large genus. Only a single female was collected from the Island of São Vicente.

***Theridionmelanostictum* O. Pickard-Cambridge, 1876**: New record for the Archipelago and found only in Santo Antão. A widespread species, with a distribution from the Mediterranean up to Japan, it is an introduction in several places in the New World.

**Family Thomisidae Sundevall, 1833**

***Runciniaflavida* (Simon, 1881)**: New record for the Archipelago and found only in Santo Antão. Widespread in Africa.

**Family Uloboridae Thorell, 1869**

***Uloboruswalckenaerius* Latreille, 1806**: New record for the Archipelago found on both Islands. An endemic species, *Uloborusrufus* Schmidt & Krause, 1995 is also known for the Archipelago. After reviewing the female genitalic characters of *U.rufus* from published information, but without checking any type material, we suspect it may be a synonymy to other species. *U.walckenaerius* has a distribution ranging from Macaronesia to Asia, also found, possibly introduced, in a coastal forest reserve in Ghana (collections of the Finnish Museum of Natural History) and in South Africa (Dippenaar-Schoeman et al. 2020).

## Figures and Tables

**Figure 4a.**
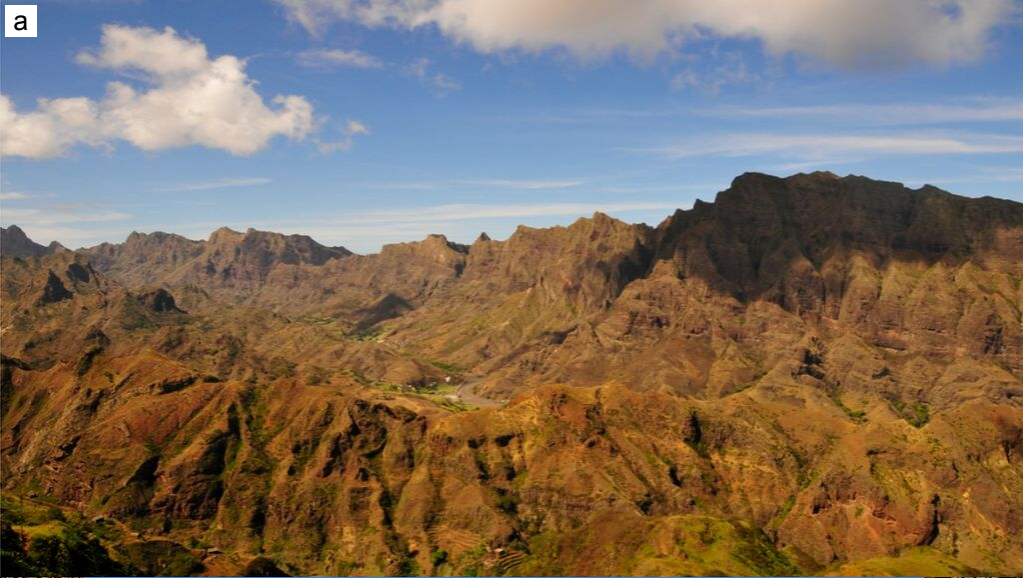
Santo Antão complex orography (Credit: Paulo A. V. Borges);

**Figure 4b.**
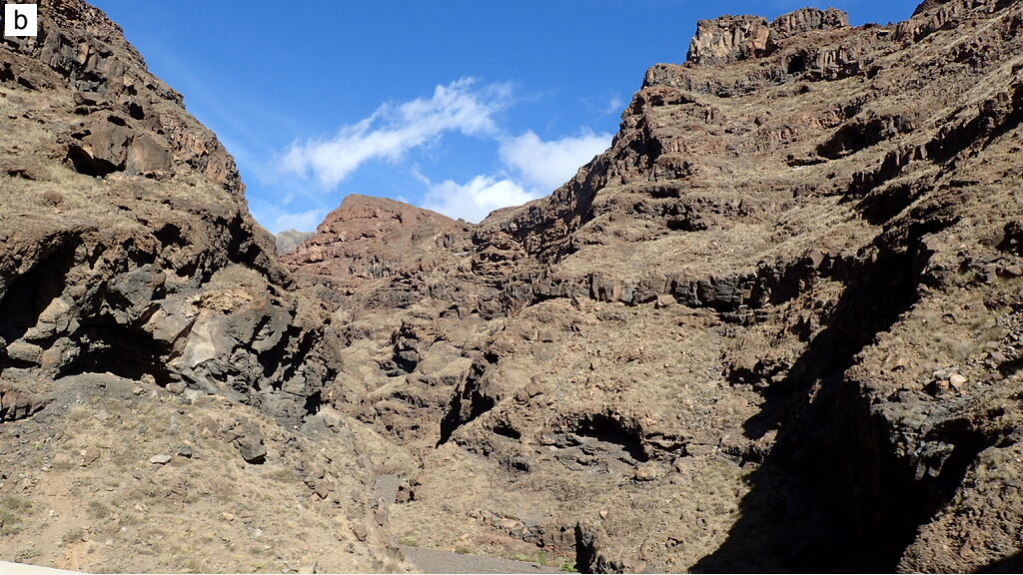
Plot 2 - Farol Fontes Pereira de Melo 2 in Santo Antão (Credit: Paulo A. V. Borges).

**Figure 6a.**
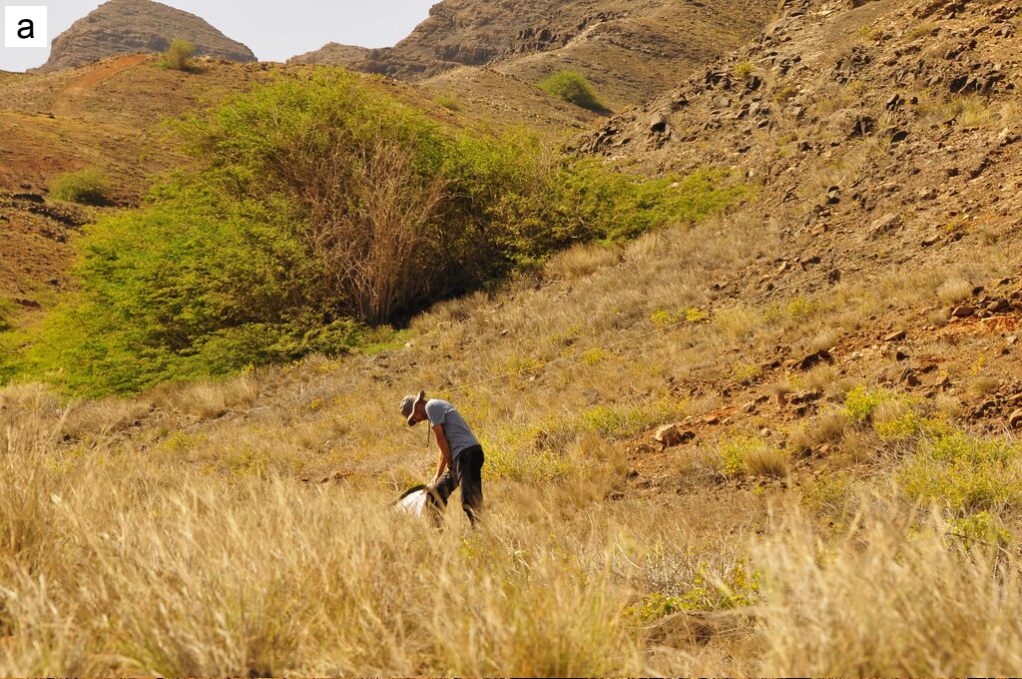
Vegetation sweeping (SW) in the plot Lazareto (Credit: Paulo A. V. Borges);

**Figure 6b.**
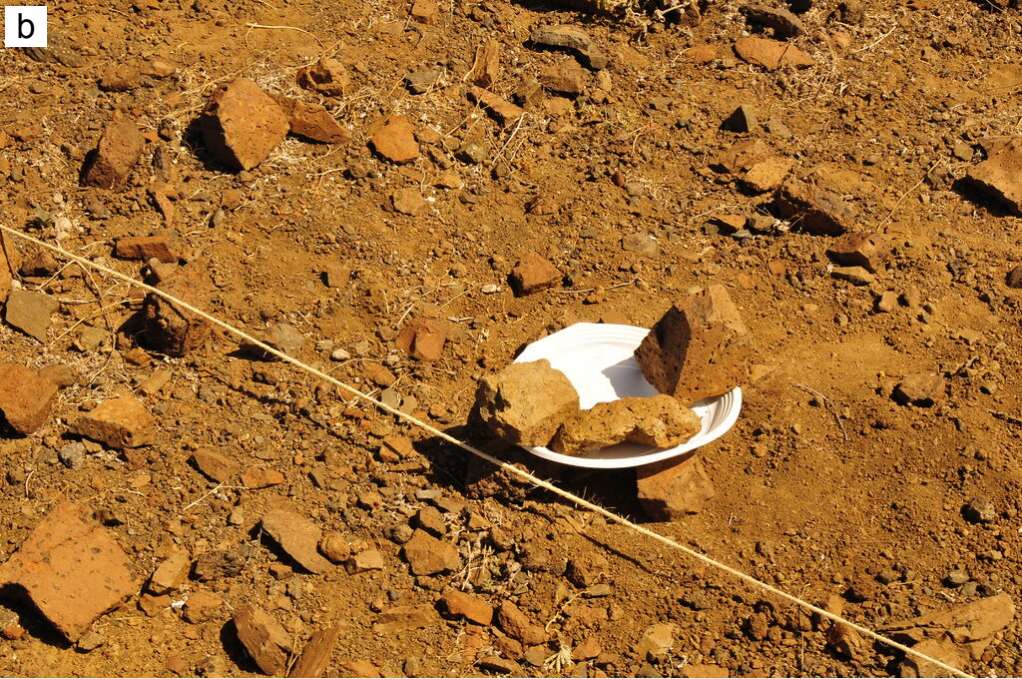
Pitfall trap with stones fixing the protection plate (Credit: Paulo A. V. Borges).

**Table 1.** List of sampling localities, including those where occasional ad-hoc sampling was performed. Code of localities as in Figs 2, 3.

**Island**	**Locality**	**Code**	**Latitude**	**Longitude**
Santo Antão	Ribeira Grande	SA-RG	17.15841	-25.07057
Santo Antão	Delgadim	SA-DG	17.13698	-25.06592
Santo Antão	Maroços	SA-MR	17.09124	-25.14153
Santo Antão	Plot 1 - Farol Fontes Pereira de Melo 1	SA-1	17.1065	-24.96802
Santo Antão	Plot 2 - Farol Fontes Pereira de Melo 2	SA-2	17.1103	-24.96801
Santo Antão	Plot 3- Chã das Furnas	SA-3	17.1697	-25.02327
Santo Antão	Plot 4 - Ribeirinha-Cruzinha	SA-4	17.1739	-25.1487
Santo Antão	Plot 5 - Ponta do Sol	SA-5	17.19314	-25.0846
São Vicente	Madeiral	SV-MD	16.82458	-24.93065
São Vicente	Monte Verde (Agave)	SV-MVA	16.87234	-24.93308
São Vicente	Monte Verde (Frigorífico)	SV-MVF	16.87286	-24.93515
São Vicente	Topo 2 Monte Verde	SV-TMV2	16.87005	-24.93289
São Vicente	Topo Monte Verde	SV-TMV	16.87025	-24.93385
São Vicente	Plot 1 - Feijoal Preto 1	SV-1	16.86288	-24.89734
São Vicente	Plot 2 - Feijoal Preto 2	SV-2	16.8632	-24.89903
São Vicente	Plot 3 - Feijoal Preto 3	SV-3	16.86178	-24.90637
São Vicente	Plot 4 - Palha Carga	SV-4	16.81482	-24.96115
São Vicente	Plot 5 - Lazareto	SV-5	16.87883	-25.02953

**Table 2.** List of identified spiders in Cabo Verde with their recorded abundance in each Island. MF - Original morphospecies code in database; STA - Santo Antão Island; VIC - São Vicente Island. New records for Cabo Verde are marked with (*).

**Family**	**Scientific Name**	**Colonisation Status**	**MF**	**STA**	**VIC**	**Total**
Araneidae	*Argiopesector* (Forsskål, 1775)	native	CV95	62	256	318
Araneidae	*Cyclosainsulana* (Costa, 1834)	native	CV21	174	49	223
Araneidae	*Cyphalonotus* sp.	native	CV89	7	0	7
Araneidae	*Cyrtophoracitricola* (Forsskål, 1775)	native	CV43	14	0	14
Araneidae	*Neosconaquincasea* Roberts, 1983 (*)	native	CV90	1	1	2
Araneidae	*Neosconasubfusca* (Koch, 1837)	native	CV11	6	11	17
Araneidae	*Pararaneusspectator* (Karsch, 1886)	native	CV180	0	1	1
Araneidae	*Trichonephilasenegalensis* (Walckenaer, 1841)	native	CV52	31	2	33
Cheiracanthiidae	*Cheiracanthiumafricanum* Lessert, 1921 (*)	native	CV60	7	0	7
Cheiracanthiidae	*Cheiracanthiumfurculatum* Karsch, 1879	native	CV65	1	0	1
Cheiracanthiidae	*Cheiracanthiumverdense* Lotz, 2011	endemic	CV131	0	123	123
Cheiracanthiidae	*Cheiracanthium* sp.	endemic	CV6	22	0	22
Cithaeronidae	*Cithaeronreimoseri* Platnick, 1991 (*)	indeterminate	CV108	0	9	9
Dictynidae	*Dictyna* sp.	indeterminate	CV4	48	53	101
Gnaphosidae	*Berlandinanigromaculata* (Blackwall, 1865)	endemic	CV19	3	23	26
Gnaphosidae	*Camillinacordifera* (Tullgren, 1910) (*)	native	CV101	2	0	2
Gnaphosidae	*Marinarozeloteslyonneti* (Audouin, 1826) (*)	introduced	CV24	16	3	19
Gnaphosidae	*Micariaignea* O. P.-Cambridge, 1872 (*)	native	CV2	44	15	59
Gnaphosidae	*Poecilochroasenilis* (O. Pickard-Cambridge, 1872) (*)	native	CV125	0	2	2
Gnaphosidae	*Scotophaeusinsularis* Berland, 1936	endemic	CV92	2	0	2
Gnaphosidae	*Setaphisatlantica* (Berland, 1936)	endemic	CV20	4	18	22
Gnaphosidae	*Smionialineatipes* (Purcell, 1908) (*)	introduced	CV102	4	0	4
Gnaphosidae	*Synaphosussyntheticus* (Chamberlin, 1924) (*)	native	CV16	2	0	2
Gnaphosidae	*Zeloteslaetus* (O. P.-Cambridge, 1872)	introduced	CV14	6	10	16
Hersiliidae	*Hersiliolaversicolor* (Blackwall, 1865)	native	CV152	0	8	8
Linyphiidae	*Agynetafuscipalpa* (C.L. Koch, 1836) (*)	introduced	CV187	2	0	2
Linyphiidae	*Metaleptyphantesperexiguus* (Simon & Fage, 1922) (*)	native	CV100	2	0	2
Linyphiidae	*Lepthyphantes* sp.	indeterminate	CV69	4	2	6
Oecobiidae	*Oecobiusmarathaus* Tikader, 1962 (*)	native	CV163	0	1	1
Oecobiidae	*Oecobiusnavus* Blackwall, 1859	introduced	CV17	26	0	26
Oecobiidae	*Oecobiuspasteuri* Berland & Millot, 1940 (*)	native	CV73	35	0	35
Oecobiidae	*Oecobiussimilis* Kulczyński, 1909 (*)	native	CV22	2	26	28
Oecobiidae	*Urocteapaivani* (Blackwall, 1868)	native	CV110	1	22	23
Oonopidae	*Khamisiahayer* Platnick & Berniker, 2015	introduced	CV159	0	4	4
Oonopidae	*Opopaeaconcolor* (Blackwall, 1859) (*)	native	CV68	5	0	5
Oonopidae	*Opopaeadeserticola* Simon, 1892 (*)	introduced	CV109	4	3	7
Oonopidae	*Opopaeaspeciosa* (Lawrence, 1952) (*)	native	CV192	0	4	4
Oonopidae	*Opopaea* sp.	indeterminate	CV193	1	0	1
Oonopidae	*Orchestina* sp.	endemic	CV9	3	18	21
Oonopidae	*Xestaspisparmata* Thorell, 1890 (*)	introduced	CV15	12	0	12
Oonopidae	Gen. indet. sp.	indeterminate	CV103	4	0	4
Oonopidae	Gen. indet. sp.	indeterminate	CV172	0	1	1
Oxyopidae	*Oxyopescaboverdensis* Schmidt & Krause, 1994	endemic	CV56	18	121	139
Oxyopidae	*Peucetiaviridis* (Blackwall, 1858)	native	CV144	0	907	907
Philodromidae	*Rhysodromuspetrobius* (Schmidt & Krause, 1995)	endemic	CV123	1	52	53
Philodromidae	*Thanatusfrederici* Denis, 1941	endemic	CV145	0	6	6
Philodromidae	*Thanatusvulgaris* Simon, 1870	native	CV133	0	6	6
Philodromidae	*Tibellusnigeriensis* Millot, 1942 (*)	native	CV54	18	0	18
Pholcidae	*Micropholcusfauroti* (Simon, 1887)	introduced	CV18	3	0	3
Pholcidae	*Modisimusculicinus* (Simon, 1893) (*)	introduced	CV76	69	0	69
Pholcidae	*Smeringopuspallidus* (Blackwall, 1858)	native	CV179	0	36	36
Prodidomidae	*Eleleisluderitz* Rodrigues & Rheims, 2020 (*)	native	CV151	0	4	4
Prodidomidae	*Prodidomus* sp.	indeterminate	CV34	1	0	1
Prodidomidae	*Prodidomus* sp.	indeterminate	CV134	0	5	5
Salticidae	*Hasariusadansoni* (Audouin, 1826)	native	CV61	14	0	14
Salticidae	*Langona* sp.	indeterminate	CV158	0	1	1
Salticidae	*Langona* sp.	indeterminate	CV194	0	1	1
Salticidae	*Neoncaboverdensis* Schmidt & Krause, 1998	endemic	CV104	2	0	2
Salticidae	*Pellenesvanharteni* Wesolowska, 1998	endemic	CV30	1	2	3
Salticidae	*Phlegrabifurcata* Schmidt & Piepho, 1994	endemic	CV121	0	5	5
Salticidae	*Phlegra* sp.	indeterminate	CV195	0	1	1
Salticidae	*Wesolowskanalymphatica* (Wesolowska, 1989)	endemic	CV167	0	5	5
Salticidae	*Wesolowskanamarginella* (Simon, 1883)	endemic	CV1	30	46	76
Salticidae	*Wesolowskana* sp.	endemic	CV190	1	0	1
Scytodidae	*Scytodesunivittata* Simon, 1882 (*)	introduced	CV46	6	0	6
Scytodidae	*Scytodesvelutina* Heineken & Lowe, 1832	native	CV191	2	0	2
Selenopidae	*Selenopsradiatus* (Latreille, 1819)	native	CV13	60	20	80
Sicariidae	*Loxoscelesrufescens* (Dufour, 1820)	native	CV77	3	0	3
Tetrablemmidae	*Tetrablemma* sp.	indeterminate	CV31	8	4	12
Theridiidae	*Argyrodesargyrodes* (Walckenaer, 1841)	native	CV91	22	11	33
Theridiidae	*Coleosomaafricanum* Schmidt & Krause, 1995	endemic	CV45	28	1	29
Theridiidae	*Enoplognathadiversa* (Blackwall, 1859) (*)	native	CV186	1	0	1
Theridiidae	*Euryopisepisinoides* (Walckenaer, 1847) (*)	introduced	CV113	0	10	10
Theridiidae	*Eurypoenatuberosa* (Wunderlich, 1987) (*)	native	CV32	8	15	23
Theridiidae	*Kochiuraaulica* (C. L. Koch, 1838)	native	CV23	2	0	2
Theridiidae	*Lasaeola* sp.	endemic	CV64	3	3	6
Theridiidae	*Paidiscuradromedaria* (Simon, 1880)	native	CV132	0	5	5
Theridiidae	*Steatodaerigoniformis* (O. Pickard-Cambridge, 1872) (*)	introduced	CV136	0	17	17
Theridiidae	*Theridionmelanostictum* O. Pickard-Cambridge, 1876 (*)	native	CV63	1	0	1
Theridiidae	*Theridion* sp.	indeterminate	CV39	7	0	7
Theridiidae	*Theridion* sp.	indeterminate	CV178	0	1	1
Theridiidae	*Tidarrencuneolatum* (Tullgren, 1910)	native	CV47	27	68	95
Thomisidae	*Misumenopsspinulosissimus* (Berland, 1936)	endemic	CV3	211	42	253
Thomisidae	*Runciniaflavida* (Simon, 1881) (*)	native	CV50	29	0	29
Thomisidae	*Thomisuscitrinellus* Simon, 1875	native	CV51	5	1	6
Thomisidae	*Thomisusonustus* Walckenaer, 1805	native	CV146	0	26	26
Uloboridae	*Uloboruswalckenaerius* Latreille, 1806 (*)	introduced	CV37	10	3	13
